# Comparison of Malated Ringer's with Two Other Balanced Crystalloid Solutions in Resuscitation of Both Severe and Moderate Hemorrhagic Shock in Rats

**DOI:** 10.1155/2015/151503

**Published:** 2015-05-27

**Authors:** Judith Keitel, Bjoern Hussmann, Sven Lendemans, Herbert de Groot, Ricarda Rohrig

**Affiliations:** ^1^Department for Trauma Surgery and Orthopaedics, University Hospital Essen, 45147 Essen, Germany; ^2^Institute of Physiological Chemistry, University Hospital Essen, Germany

## Abstract

In preclinical treatment of polytraumatized patients crystalloids are preferentially used. To avoid metabolic acidosis, metabolizable anions like lactate or acetate are used to replace chloride in these solutions. We here studied the effects of malated Ringer's in resuscitation of both shock severities in comparison to lactated and acetated Ringer's. Male Wistar rats underwent severe (mean arterial blood pressure (MAP) of 25–30 mmHg) or moderate (MAP 40–45 mmHg) hemorrhagic shock. Adjacent to the shock period animals were resuscitated with acetated (AR), lactated (LR), or malated Ringer's (MR) and observed for 150 min. MR improved survival compared with LR and AR in severe hemorrhagic shock whereas it was equally effective to LR and superior to AR in moderate hemorrhagic shock. In all other parameters tested, MR was also effective similar to the other solutions under these conditions. We conclude that MR is preferable to AR and LR in resuscitation of hemorrhagic shock independent of shock depth. The positive effects of MR may stem from the absence of any adverse impact on energy metabolism under both conditions.

## 1. Introduction

Beside craniocerebral injury, hemorrhagic shock is one of the main causes of death among trauma patients [1, 2]. Although the resuscitation regimes changed over the past decades, the first priority is still the control of the bleeding source and the immediate restoration of the lost blood volume. In initial preclinical treatment of polytraumatized patients, crystalloids are preferentially used. Because hemorrhagic shock is strongly correlated with the occurrence of metabolic acidosis, the infusion of high amounts of chloride is considered to be counter-indicated [3]. Thus, in the resuscitation fluids chloride is partially substituted by metabolizable anions such as lactate, acetate, or malate.

During the metabolism of lactate, acetate, and malate, H^+^ ions are consumed (or HCO_3_^−^ ions generated) supporting a normalization of blood pH. Furthermore, these anions may improve energy supply to vital organs such as the heart. For lactate, the alkalizing effect was first shown by Hartmann and later confirmed by several other groups [3–5]. We could recently demonstrate that lactate infusion may be detrimental when used in resuscitation of a severe hemorrhagic shock in rats whereas it even may prolong survival when administered in a more moderate form of hemorrhagic shock [6, 7].

In order to replace lactate, acetate can be used as metabolizable anion [8]. It is ubiquitously metabolized and there are no negative side effects known for the application of acetate in resuscitation solutions in severe hemorrhagic shock [9, 10]. However, some isotonic crystalloid solutions used in hemodialysis containing acetate are assumed to mediate vasodilatory actions [11]. Although the presumption about vasodilation could not be confirmed in severe hemorrhagic shock by our group [10], there are still some efforts to find a resuscitation solution with more protective properties. Malate seems to be a promising candidate not only in treatment of metabolic acidosis but also in the maintenance of energy supply during hypoxic conditions [12–14]. Like acetate, it is ubiquitously metabolized and holds a pivotal role in intermediary metabolism, that is, in the tricarboxylic acid (TCA) cycle and the malate-aspartate-shuttle. However, the information about the effects of malate in direct comparison to lactate and acetate especially in the resuscitation of severe hemorrhagic shock is scarce. There is only one study comparing malated Ringer's with lactated Ringer's and normal saline in a moderate form of hemorrhagic shock [15]. Therefore, we here study the effects of lactated Ringer's (LR), acetated Ringer's (AR), and malated Ringer's (MR) in the resuscitation from a severe (25–30 mmHg) and a moderate form (40–45 mmHg) of hemorrhagic shock on survival, acid-base status, and other parameters in rats.

## 2. Methods

### 2.1. Chemicals/Materials

Normal saline and AR were from B. Braun (Melsungen, Germany) and MR, Ringer's solution (RS), and LR (only containing l-lactate) from Fresenius (Bad Homburg, Germany). Ketamine 10% was from Ceva (Düsseldorf, Germany), lidocaine (Xylocaine 1%) from AstraZeneca (Wedel, Germany), and acid citrate dextrose A solution from Baxter (Deerfield, IL). Portex catheters (inner diameter: 0.58 mm, outer diameter: 0.96 mm; Smiths Medical International, Hythe, UK) and medical oxygen (Air Liquide, Düsseldorf, Germany) were obtained from the vendors listed.

### 2.2. Animals

Male Wistar rats (400–500 g) were obtained from the central animal unit of the Essen University Hospital. Animals were kept under standardized conditions of temperature (22°C ± 1°C), humidity (55% ± 5%), and 12 h/12 h light-dark cycles. The rats had free access to water, were fed with standard chow (Ssniff-Spezialditäten, Soest, Germany), and were not fasted before the experiments. All animals received human care according the standards of Annex III of the directive 2010/63/EU of the European Parliament and of the Council of 22 September 2010 on the protection of animals used for scientific purposes [16]. The experimental protocol has been approved by the North Rhine-Westfalia State Office for Nature, Environment and Consumer Protection, Recklinghausen, Germany (Landesamt für Natur, Umwelt und Verbraucherschutz Nordrhein Westfalen, Recklinghausen, Deutschland), based on the local animal protection act.

### 2.3. Anesthesia, Analgesia, and Surgical Procedures

Anesthesia, analgesia, catheter insertions, shock induction, resuscitation schedule, and blood sampling, were basically performed as described previously with slight modifications [10]. Rats were anesthetized with isoflurane (2% in 100% medical O_2_ at 4 L/min for induction of anesthesia, 1%–1.5% at 2 L/min throughout the experiment) through face masks connected to a vaporizer (Isofluran Vet. med. Vapor; Dräger, Lübeck, Germany) and received ketamine (50 mg/kg, s.c.) into the right chest wall for analgesia. Lidocaine (5 mg/kg, s.c.) was administered before a skin-deep incision along the right groin. Subsequently, a Portex catheter was placed within the femoral artery and the femoral vein. Each catheter was fixed with surgical suture.

### 2.4. Induction of Hemorrhagic Shock and Resuscitation Regimen

After insertion of the catheter, animals were allowed to adapt for 20 min before hemorrhagic shock was induced by removing 1-2 mL blood every 3 min through the femoral artery catheter using 2-mL syringes (Terumo, Leuven, Belgium). Bleeding was continued until the mean arterial blood pressure (MAP) dropped either to 25–30 mmHg for severe shock or to 40–45 mmHg for moderate shock, which typically took about 20 min in both groups. During the following 10 min, the MAP was fine-tuned by sampling of smaller blood volumes (0.5–1 mL). For the next 60 min, the MAP was kept between 25–30 mmHg and 40–45 mmHg, respectively, typically without the need of any further intervention. In some individual cases small blood samples had to be withdrawn or administered to keep the MAP in the desired range. The blood used for adjustment was collected during shock induction in syringes containing acid dextrose A solution (ACD-A). After the shock phase, study group-specific resuscitation fluids were infused into the femoral vein within 30 min using a syringe pump (Perfusor-Secura FT; B. Braun, Melsungen, Germany). Experiments were continued for another 150 min, unless the animals died earlier. To compensate for fluid loss over surgical areas and the respiratory epithelium, RS (5 mL/kg/h) was infused through the femoral vein catheter throughout the experiment.

### 2.5. Experimental Groups

All 46 animals were randomly assigned to the following groups:

- sham group (no shock, no resuscitation, six animals),
- moderate shock/AR group (shock, resuscitation with acetated Ringer's equal to three times the shed blood volume, eight animals),
- moderate shock/LR group (shock, resuscitation with lactated Ringer's equal to three times the shed blood volume, eight animals),
- moderate shock/MR group (shock, resuscitation with malated Ringer's equal to three times the shed blood volume, eight animals),
- severe shock/AR group (shock, resuscitation with acetated Ringer's equal to three times the shed blood volume, eight animals),
- severe shock/LR group (shock, resuscitation with lactated Ringer's equal to three times the shed blood volume, eight animals),
- severe shock/MR group (shock, resuscitation with malated Ringer's equal to three times the shed blood volume, eight animals).

The volume of fluid to be used for resuscitation is based on the well established 3 : 1 rule [17] taking into consideration the short intravasal half-life of crystalloid solutions.

### 2.6. Biomonitoring

Systolic blood pressure, diastolic blood pressure, and MAP were recorded continuously via the femoral artery catheter that was connected to a pressure transducer and displayed on a monitor. Heart rates were determined from systolic blood pressure spikes. The core body temperature of the rats was kept at 37.5 ± 0.5°C and continuously monitored via a rectal sensor. Cooling of the animals was prevented by a cover with aluminum foil and by means of an underlying heated operating table. The oxygen saturation was recorded continuously using a pulse oximeter (OxiCliq A; Nellcor, Boulder, CO, USA) placed at the left hind limb. The breathing rate was determined based on the ventilation movements in 15 sec. All biomonitoring parameters were recorded in 10 min intervals.

### 2.7. Assessment of Blood and Plasma Parameters

Using a 2-mL syringes containing 80 IU electrolyte-balanced heparin (Pico50; Radiometer Medical, Brønshøj, Denmark), blood samples (0.5–0.7 mL) for blood gas analysis and the assessment of marker enzymes activities were taken from the femoral artery immediately after its insertion (*T* = 0 min), before shock induction (*T* = 30 min), after the end of shock induction (*T* = 60 min), immediately before the beginning of resuscitation (*T* = 120 min), at the end of resuscitation (*T* = 150 min), and during the final observation phase (at* T* = 180, 240, and 300 min). For each blood sampling, animals were substituted with a 0.5 mL bolus of RS via the femoral artery to keep the catheter functional. Arterial blood pH, oxygen, and carbon dioxide partial pressure (pO_2_, pCO_2_), oxygen saturation, base excess (BE), hematocrit, electrolytes (Na^+^, K^+^, Cl^−^, Ca^2+^), osmolarity, and lactate and glucose concentration were assessed with a blood gas analyzer (ABL 715; Radiometer, Copenhagen, Denmark). Blood plasma was obtained by centrifugation (3,000 ×g for 15 min at 25°C) and stored at 4°C until its use (within 4 h). Plasma activities of lactate dehydrogenase (LDH) as a general marker for cell injury, creatine kinase (CK) as a marker for muscle cell injury, and glutamate-pyruvate transaminase (GPT) and glutamate-oxaloacetate transaminase (GOT) as markers of liver injury were determined with a fully automated clinical chemistry analyzer (Vitalab Selectra E; VWR International, Darmstadt, Germany).

### 2.8. Statistical Analysis

Experiments were performed with eight animals per experimental group, except for the sham group, which consisted of six animals. Data are expressed as mean values ± SEM. Outliers were removed after box-plot analysis. Comparisons among multiple groups were performed using one-way analysis of variance (ANOVA) either for nonrecurring or for repeated measures followed by Fisher (LSD, least significant difference) post hoc analysis. Survival curves were generated according to the Kaplan-Meier method and were compared with the log-rank test. *P* < 0.05 was considered significant.

## 3. Results

### 3.1. Survival

**Figure 1:**
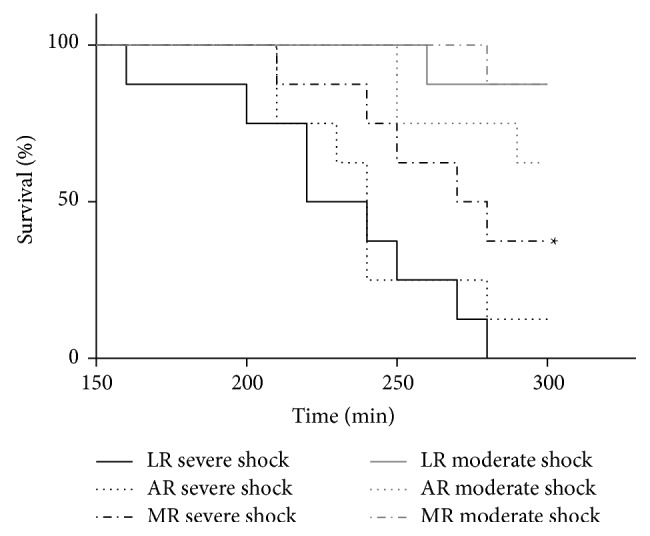
Effects of lactated, acetated, and malated Ringer's on survival in severely/moderately shocked rats. Rats underwent severe and moderate hemorrhage and then were resuscitated with either lactated Ringer's (LR), acetated Ringer's (AR), or malated Ringer's solution (MR). The survival per group is shown as Kaplan-Meier plot (*n* = 7-8 animals; log-rank ^*^ *P* < 0.05 (versus severe shock/AR and LR)). Note: the time point at which the first animal among the shock groups died (e.g., *T* = 160 min for the severe shock/LR group) was defined as the last one where measurements between the respective groups were compared.

All animals of the sham group survived the experiment (data not shown). In the severe shock groups, the animals receiving LR were the first who died (beginning at* T* = 160 min; the last one died 30 min before the end of the experiment at* T* = 270 min; Figure 1). Although one animal of the severe shock/AR group survived the whole experimental time, the median survival of the shock/LR and the shock/AR group was similar (230 versus 240 min). In marked contrast, resuscitation with MR significantly prolonged survival (first rat died at* T* = 210 min, median survival 275 min) compared to LR. Three animals of this group survived until the end of the experiment.

In the moderate shock/MR and LR group, only one animal died during the experiment (at* T* = 280 min and* T* = 260 min, resp.). Three of the moderate shock/AR group rats died before the end of the experiment (two animals at* T* = 250 min and one at* T* = 290 min).

### 3.2. Mean Arterial Blood Pressure

**Figure 2:**
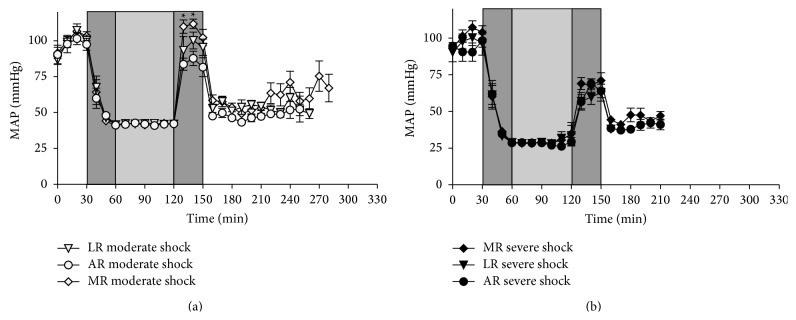
Effects of lactated, acetated, and malated Ringer's on MAP in severely/moderately shocked rats. Rats underwent moderate and severe hemorrhage (shock induction: dark grey; shock phase: light grey) and then were resuscitated (dark grey) with either lactated Ringer's (LR), acetated Ringer's (AR), or malated Ringer's solution (MR) and observed for further 150 min or until the first animal of the respective group died. (a) Mean arterial blood pressure (MAP) in moderate hemorrhagic shock of LR, AR, and MR and (b) MAP in severe hemorrhagic shock of LR, AR, and MR treated animals. Shown are mean values ± SEM (*n* = 8 animals per shock group; ^*^ *P* < 0.05 (versus moderate shock/AR and LR)). SEM values not visible are hidden by the symbols.

In the sham group animals the mean arterial blood pressure (MAP) remained fairly constant at 100 mmHg throughout the experiment (data not shown). In the animals of the shock groups, the MAP was decreased either to 25–30 mmHg (severe shock) or to 40–45 mmHg (moderate shock) within 30 min and subsequently maintained in these ranges for 60 min (Figure 2). In the severe shock groups, upon resuscitation, the MAP increased to values around 70 mmHg; subsequently, in the postresuscitation phase it rapidly decreased to about 40–45 mmHg without significant differences between the groups. Upon resuscitation, the MAP of the moderate shock/MR group recovered to about 110 mmHg whereas it increased only to about 95 and 85 mmHg in the moderate shock/LR and AR group, respectively. In all moderate shock groups, the MAP declined to around 50–65 mmHg in the postresuscitation phase.

### 3.3. Hematocrit and Plasma Electrolyte Concentrations

In the sham group animals, no significant changes in hematocrit and plasma electrolyte concentrations were observed (data not shown). In all shock groups, independent of shock depth, there were no alterations in the Na^+^ and Ca^2+^ concentrations, but slight elevations in the Cl^−^ and K^+^ concentrations during the postresuscitation phase. In contrast to the moderate shock groups where the hematocrit remained stable during the postresuscitation phase, in the severe shock groups it slightly increased after resuscitation.

### 3.4. Acid-Base Status

**Figure 3:**
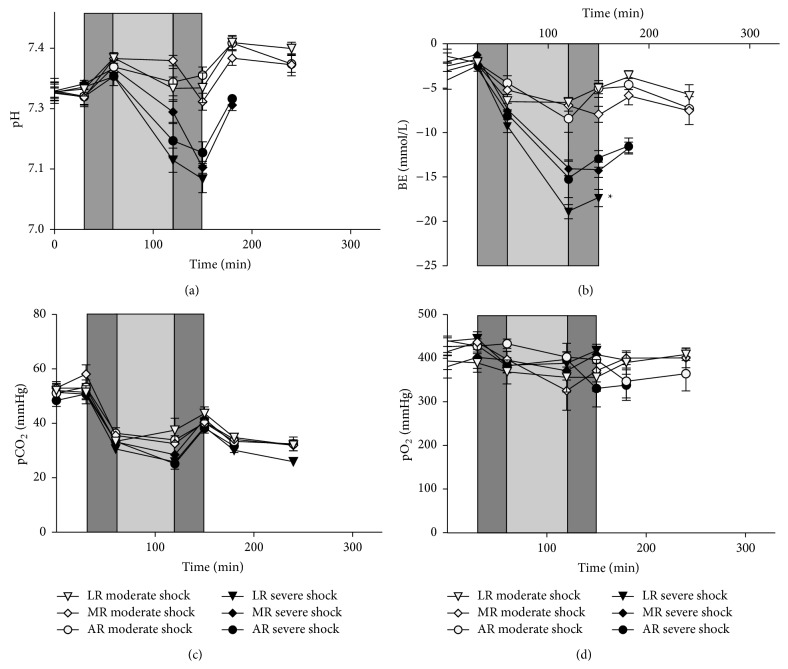
Effects of lactated, acetated, and malated Ringer's on the acid-base balance in severely/moderately shocked rats. Rats underwent severe and moderate hemorrhage (shock induction: dark grey; shock phase: light grey) and then were resuscitated (dark grey) with either lactated Ringer's (LR), acetated Ringer's (AR), or malated Ringer's solution (MR) and observed for further 150 min or until the first animal of the respective group died. (a) Blood pH, (b) base excess, (c) CO_2_ partial pressure (pCO_2_), and (d) O_2_ partial pressure (pO_2_). Values are means ± SEM (*n* = 8 animals per shock group; ^*^ *P* < 0.05 (versus severe shock/AR)). SEM values not visible are hidden by the symbols.

The pH, BE, and pCO_2_ of the sham group were stable at values of 7.35, −3 mmol/L, and 45 mmHg, respectively, throughout the experiment (data not shown). During shock induction, the shock and the resuscitation phase, the pH decreased to values around 7.1 in the severe shock group animals, but remained around 7.35 in the moderate shock groups (Figure 3(a)). In the postresuscitation phase, the pH increased somewhat to values around 7.3 in the severe shock groups and to around 7.4 in the moderate shock groups. During shock induction and the shock phase, the BE rapidly decreased to around −18 mmol/L (severe shock/LR group), −15 mmol/L (severe shock/MR and AR group), and −6 mmol/L (moderate shock groups), respectively, and varied around these values during the resuscitation and the postresuscitation phase (Figure 3(b)). In all shock groups, the pCO_2_ rapidly decreased during shock induction to around 35 mmHg, remained around this value in the shock phase, regained 40 mmHg at the end of the resuscitation phase, but subsequently decreased to about 30 mmHg (Figure 3(c)). The pO_2_ varied around 400 mmHg in all groups studied during the whole experiment (Figure 3(d)).

### 3.5. Lactate and Glucose Concentrations

**Figure 4:**
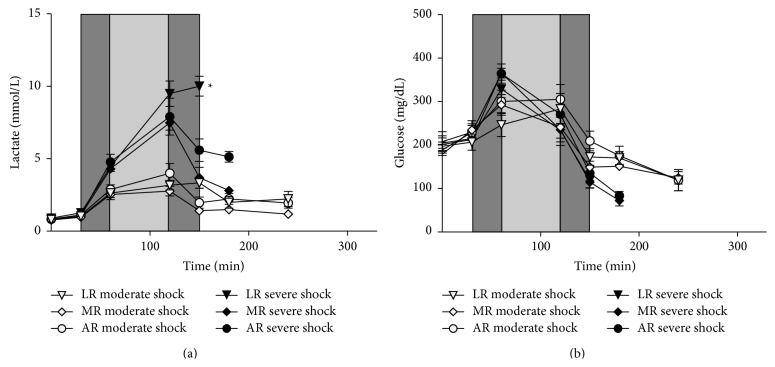
Effects of lactated, acetated, and malated Ringer's on lactate and glucose concentration in severely/moderately shocked rats. Rats underwent severe and moderate hemorrhage (shock induction: dark grey; shock phase: light grey) and then were resuscitated (dark grey) with either lactated Ringer's (LR), acetated Ringer's (AR), or malated Ringer's solution (MR) and observed for further 150 min or until the first animal of the respective group died. Blood glucose (a) and lactate concentrations (b). Values are means ± SEM (*n* = 8 animals per shock group; ^*^ *P* < 0.05 (versus severe shock/LR)). SEM values not visible are hidden by the symbols.

Sham group plasma lactate and glucose concentration remained stable at around 1 mmol/L and 200 mg/dL, respectively, during the whole experiment (data not shown). In the severe shock groups, the plasma lactate concentration increased to about 8 mmol/L during shock induction and the shock phase. During resuscitation and the postresuscitation phase it either remained at this level (severe shock/LR group) or declined to about 5 (severe shock/AR group) and 3 mmol/L (severe shock/MR group), respectively. In all moderate shock groups, the plasma lactate concentration rose to 2 to 3 mmol/L during shock induction and the shock phase but subsequently decreased to about 2 mmol/L (Figure 4(a)). The blood glucose concentration rapidly increased during shock induction in all shock groups to about 350 (severe shock) and 250 mg/dL (moderate shock; Figure 4(b)), respectively. During shock, it varied around 250 mg/dL independent of shock depth and subsequently decreased during the resuscitation and the postresuscitation phase, finally reaching values of approximately 80 mg/dL (severe shock) and 100 mg/dL (moderate shock), respectively.

### 3.6. Plasma Enzyme Activities

**Figure 5:**
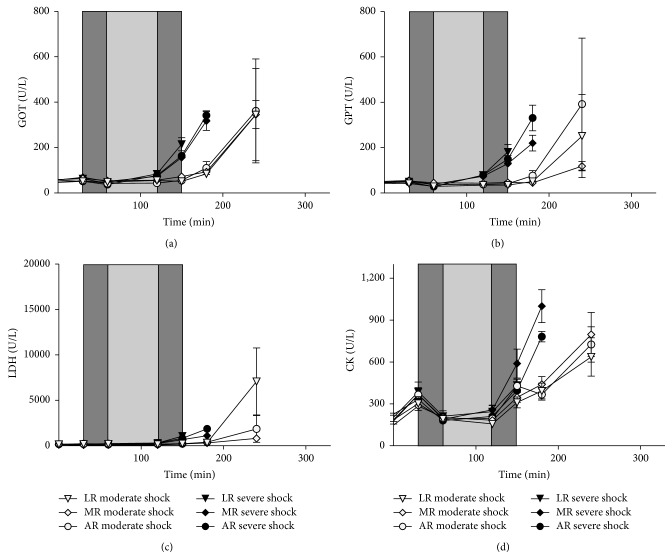
Effects of lactated, acetated, and malated Ringer's on plasma enzyme activities in severely/moderately shocked rats. Rats underwent severe and moderate hemorrhage (shock induction: dark grey; shock phase: light grey) and then were resuscitated (dark grey) with either lactated Ringer's (LR), acetated Ringer's (AR), or malated Ringer's solution (MR) and observed for further 150 min or until the first animal of the respective group died. GOT activity (a), GPT activity (b), LDH activity (c), and CK activity (d). Values are means ± SEM (*n* = 8 animals per shock group); SEM values not visible are hidden by the symbols.

In the sham group, plasma GOT, GPT, LDH and CK activities did not exceed 80 U/L, 60 U/L, 160 U/L, and 350 U/L, respectively, throughout the whole experiment (data not shown), in order to match the figure. Plasma enzyme activities began to rise upon resuscitation and in the postresuscitation phase without any differences within the groups of one shock depth (Figure 5). The activities of the moderate shock groups, however, exhibited a delayed increase in comparison with the severe shock groups.

## 4. Discussion

At present, the most frequently applied metabolizable anion in crystalloid solutions is lactate. LR was shown to be effective in the treatment of metabolic disorders in children [4] and it provided an improved cardiac performance and ameliorated outcome in the treatment of hemorrhagic shock when compared with normal saline [3, 18, 19]. In our hands, however, LR was superior to pure Ringer's solution in the resuscitation of a moderate form of hemorrhagic shock, but exhibited clearly detrimental effects in resuscitation of severe hemorrhagic shock [6, 7, 10]. Similar results were obtained here. In severe hemorrhagic shock, the shortest survival time was found in those animals resuscitated with LR (Figure 1), while in moderate hemorrhagic shock resuscitation with LR even slightly improved survival and to some extent increased MAP (Figure 2) as compared with AR. This opposing behaviour of LR upon resuscitation following severe or moderate hemorrhagic shock may be best explained by differences in lactate catabolism under both conditions. Following severe hemorrhagic shock, administration of LR provides an additional lactate burden (increase in lactate concentration; see also Figure 4), which may result in an inhibition of glycolysis and thus energy production [7, 20–22]. In contrast, after moderate hemorrhagic shock lactate is readily metabolized, as also indicated by the improved blood pH under these conditions (Figure 3), and thus may especially ameliorate cardiac function [6, 19].

For acetate supplied in saline solutions, mainly beneficial effects have been described, such as a positive influence on the acid-base balance in and after hemorrhagic shock, presumably resulting from its fast and ubiquitous catabolic degradation [23–25]. We recently showed that AR was superior to LR in severe hemorrhagic shock in rats [10]. In line with these results, in the present study in severe hemorrhagic shock AR tended to prolong survival and clearly improved acid-base status as compared to LR (Figures 3 and 4). In moderate hemorrhagic shock, however, AR appears to shorten survival as compared with both other solutions (Figure 1) and resuscitation with AR resulted in a smaller increase in MAP as compared with LR and especially with MR (Figure 2). An explanation for this unexpected behavior might be that acetate is a less appropriate substrate, especially of the heart, as compared to malate and lactate under these conditions.

Although the metabolism of malate and its effects on metabolic acidosis and the cardiovascular system under hypoxic conditions have been extensively studied [12, 13, 26–28], there are only a few studies dealing with its efficacy in the treatment of hemorrhagic shock [14, 15]. In the study of Dai et al., where a moderate form of hemorrhagic shock was applied (40 mmHg, as in the moderate hemorrhagic shock of the present experiments), remarkable improvements in MAP, improved acid-base status, and reduced organ injury were shown in male Sprague-Dawley rats treated with a self-established Ringer's malate solution as compared with Ringer's lactate and normal saline [15]. In the present study, comparable results were obtained in moderate hemorrhagic shock. Even more impressive, however, resuscitation with MR led to an increased survival in severely shocked animals in relation to both LR and AR (Figure 1). Furthermore, MR somewhat improved acid-base balance as compared to LR under these conditions (Figure 3). This superiority of MR is most likely related to malate's pivotal role in the intermediary metabolism. Under aerobic conditions, malate is readily oxidized to oxaloacetate via the tricarboxylic acid cycle thereby exerting anaplerotic functions to fuel ATP synthesis [29]. It is also part of a shuttling system in the inner mitochondrial membrane, the malate-aspartate shuttle, ensuring the transport of reduction equivalents generated in the cytosol into the mitochondrion [30, 31]. It is well known that the malate-aspartate shuttle decreases cytosolic NADH/NAD^+^ ratio and thus may support oxidative phosphorylation and glycolysis, a mechanism which may even work under hypoxic (but not anoxic) conditions [12, 32, 33]. Moreover, in hypoxia malate may stimulate substrate-level phosphorylation by partly inverted reactions of the tricarboxylic acid cycle [34–36]. Beside its effects on energy metabolism, malate may also exert antioxidative properties due to providing reduction equivalents for the elimination of reactive oxygen species [15].

## 5. Conclusions

The present study indicates that the successful application of LR and AR in the treatment of hemorrhagic shock is dependent on shock severity. While LR provided almost optimal results in moderate hemorrhagic shock, AR seems more advantageous in severe hemorrhagic shock. Independent of shock severity, however, MR appears to be the superior resuscitation solution. In moderate hemorrhagic shock it provided results comparable to LR and in severe hemorrhagic shock outcome was clearly improved as compared to both other solutions. Thus, the use of MR, especially in preclinical resuscitation schedules in which the magnitude of shock can only be estimated, should be preferred. However, the results presented here apply only for the experimental conditions used here, that is, severe and moderate hemorrhagic shock with a controlled blood loss but without further attempts at treatment. Whether they can be adopted to the treatment of polytraumatized patients remains to be elucidated.

## Abbreviations

ACD-A: Acid citrate dextrose-A
ANOVA: Analysis of variance
AR: Acetated Ringer's
BE: Base excess
bpm: Beats per min
CK: Creatine kinase
GOT: Glutamate-oxaloacetate transaminase
GPT: Glutamate-pyruvate transaminase
Hct: Hematocrit
LDH: Lactate dehydrogenase
LR: Lactated Ringer's
MAP: Mean arterial blood pressure
MR: Malated Ringer's
pCO_2_: Carbon dioxide partial pressure
pO_2_: Oxygen partial pressure.
